# Development of Personal Protective Clothing for Reducing Exposure to Insecticides in Pesticide Applicators

**DOI:** 10.3390/ijerph17093303

**Published:** 2020-05-09

**Authors:** Manoch Naksata, Anucha Watcharapasorn, Surat Hongsibsong, Ratana Sapbamrer

**Affiliations:** 1 Department of Physics and Material Science, Faculty of Science, Chiang Mai University, 239, Huay Kaew Road, Suthep Subdistrict, Muang District, Chiang Mai 50200, Thailand; manochnak@gmail.com (M.N.); anucha@stanfordalumni.org (A.W.); 2 Research Center in Physics and Astronomy, Faculty of Science, Chiang Mai University, Chiang Mai 50200, Thailand; 3 School of Health Sciences Research, Research Institute for Health Sciences, Chiang Mai University, 110 Inthavaroros Road, Sriphum Subdistrict, Muang District, Chiang Mai 50200, Thailand; 4 Department of Community Medicine, Faculty of Medicine, Chiang Mai University, 110 Inthavaroros Road, Sri Phum Subdistrict, Muang District, Chiang Mai 50200, Thailand

**Keywords:** personal protective equipment, personal protective clothing, fabric, pesticide, insecticide, chlorpyrifos, cypermethrin, safety, risk reduction

## Abstract

Wearing appropriate personal protective equipment during the application of pesticides is one method of reducing dermal exposure to pesticides. Thus, the aim of this research is to develop personal protective clothing (PPC) coated with gum rosin and investigate the efficiency of its level of protection against chlorpyrifos and cypermethrin. Comparison of the protection efficiency of each PPC with Tychem^®^ C coveralls was also investigated. Five commercially available cotton fabrics were chosen for tailoring the PPC, and then, the PPC was coated with a gum rosin finish to provide water repellence. The efficiency of the level of protection of the gum rosin-coated PPC against insecticides was tested in a laboratory (closed chamber). The remarkable findings were that the % protection efficiencies for all the PPC, with the exception of one, were not significantly different to those for Tychem^®^ C coveralls. The protection efficiencies ranged from 99.85% to 99.97% against chlorpyrifos and 99.11% to 99.89% against cypermethrin. Therefore, our results suggest that gum rosin-coated clothing provided satisfactory levels of protection against insecticides and could be considered as suitable protective clothing for pesticide applicators. Choice of an appropriate fabric for coating with gum rosin also needs to be considered. A further study in field conditions is warranted to confirm the protection efficiency in a working environment.

## 1. Introduction

It has been known that pesticides are associated with acute and chronic health effects in agricultural workers who are exposed to them. Exposure to pesticides can potentially occur during mixing, loading, application of the pesticides, and other activities in farms. Due to pesticide application taking up the highest proportion of the working time in comparison to the tasks of mixing and loading, exposure during pesticide application might be more significant [1,2]. The primary route of exposure to pesticides is dermal contact, while exposure through inhalation is rather limited, due to the low vapor pressures of some pesticides [3,4,5]. Although national and international sectors have made an effort to reduce pesticide exposure in agricultural workers by laying down regulation and legislations, the implementation of engineering and system controls is difficult for many [6]. Therefore, wearing appropriate personal protective equipment in all activities associated with pesticide handling is one measure to reduce dermal exposure to the toxic chemicals involved [6,7].

With regard to personal protective clothing (PPC), clothing made of non-woven synthetic materials which are coated in certain chemicals to make it non-porous has the highest levels of protection against pesticides [8,9,10]. A category III type 3 partial body gown (Tychem^®^ F Gown style) had a protective effect of 98.7%, while Tyvek coveralls had a protective effect higher than 97% [8,9]. However, these items of PPC are quite expensive, so consequently, most agricultural workers cannot afford them [11,12,13]. These items of PPC can also interfere with heat exchange, causing discomfort and development of heat stress under hot and humid conditions [14,15]. Therefore, the high price and discomfort of these PPC are the main cited reasons agricultural workers do not use them when working with pesticides [11]. A systematic review by Sapbamrer and Thammachai [16] stated that a long-sleeved shirt and long-sleeved trousers were the most basic PPE worn among pesticide handlers across the world. Most agricultural workers wore work clothing made of woven fabric when working on farms, resulting in the easy penetration of pesticides through to the skin [1,17]. Pesticide penetration through woven fabric depends on type of pesticide, fabric thickness, fabric weight, yarn twist, and fabric composition [18,19,20,21].

The outcomes of some studies have facilitated the development of woven fabrics coated with certain chemicals (including polyurethane, rubber, teflon, and fluorocarbon) to protect against pesticides and simultaneously confer better thermal comfort [22,23,24,25]. The study by Naksata and Naksata (Petty Patent no. 7450, 8 July 2010, Thailand) also reported on the efficacy of a novel cotton fabric coated with gum rosin to provide water repellence [26]. Cotton fabric has numerous pores in a woven structure and can absorb large amounts of liquid including sweat, leading to wearers feeling more comfortable in hot conditions [27]. Gum rosin is a natural resin, which is extracted from pine trees. It is usually used in its crude raw form for the production of soap, varnishes, sealing wax, and various adhesives [28]. The cotton which was coated with gum rosin showed a water resistance for longer than 24 hours and a contact angle of water repellency greater than 120° [26]. We expect that the gum rosin-coated clothing can protect against pesticides and make wearers comfortable when applying pesticides. This study focuses on insecticides, which are the most extensively used pesticides in agriculture worldwide, our main focus being chlorpyrifos and cypermethrin. Thus, the aim of this research is to develop PPC which is made of cotton coated with gum rosin and investigate the efficiency of the clothing in the level of protection given against chlorpyrifos and cypermethrin. Comparison of the protection efficiency of each PPC with Tychem^®^ C coveralls was also investigated.

## 2. Materials and Methods 

### 2.1. Coating of Personal Protective Clothing with Gum Rosin

**Figure 1:**
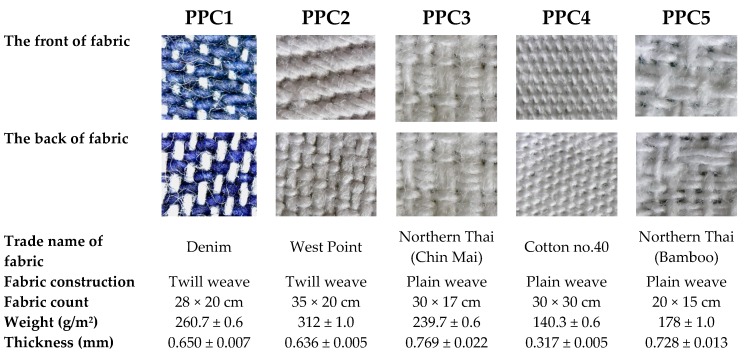
Properties of fabrics which were used to tailored personal protective clothing (PPC).

Five commercially available cotton fabrics (PPC1–PPC5) were chosen for the making of the PPC. The five fabrics were selected because they gave the highest level of protection against pesticides in a preliminary laboratory investigation. Fabric construction, fabric count, weight, and thickness of each cotton fabric were measured (Figure 1). Fabric count and weight were measured in accordance with ASTM D3775 and ASTM 3776, respectively. Fabric thickness was measured in accordance with ASTM D1777–96. A Tychem^®^ C coverall (DuPont, Vietnam) was also used in the study for comparison of the level of protective efficiency with the PPC in our study. The PPC was made into coveralls of a similar size and pattern as the Tychem^®^ C coverall. The coveralls were tailored to fit the manikin used in the tests. 

The PPC was coated with gum rosin finish to provide water repellence. Physical and chemical characteristics of gum rosin are as follows: pale yellow to amber fragments, molecular formula C_20_H_30_O_2_, molecular weight 302.5 g/mole, density 1.07 g/cm^3^, melting point 100–150 °C, and flash point 187 °C [29]. The coating process was completed following the method described by Naksata and Naksata (Petty Patent no.7450, 8 July 2010, Thailand) [26]. Prior to the coating process, the coveralls were laundered with commercially available detergent and tap water and dried at 60–80 °C. They were then soaked in gum rosin solution 1–3% w/v (M/L = 1:20) (Chemwinfo Co., Ltd, Bangkok, Thailand) for 15 min, and potassium aluminum sulfate 1–3% w/v (M/L = 1:20) (World Chemical Co., Ltd, Chiang Mai province, Thailand) for 15 min. After the coating process, the coveralls were spin-dried at 3000 rounds per min for 3 min and dried at 60–80 °C.

### 2.2. Test Insecticides

Two insecticides, including chlorpyrifos 40% w/v, emulsifiable concentrate (Tradename: Cosmic 40, ICP Ladda Co.,Ltd, Thailand), and cypermethrin 35% w/v, emulsifiable concentrate (Tradename: Thaiperthroid 35, Pato Chemical Co., Ltd, Bangkok, Thailand), were used in the study. Before the experiments the insecticides were diluted to a final concentration of 2 mL/1 L of water, which is the recommended dilution on the labels on the insecticides.

### 2.3. Closed Chamber Testing of the Protective Efficiency of the Gum Rosin-Coated PPC

**Figure 2:**
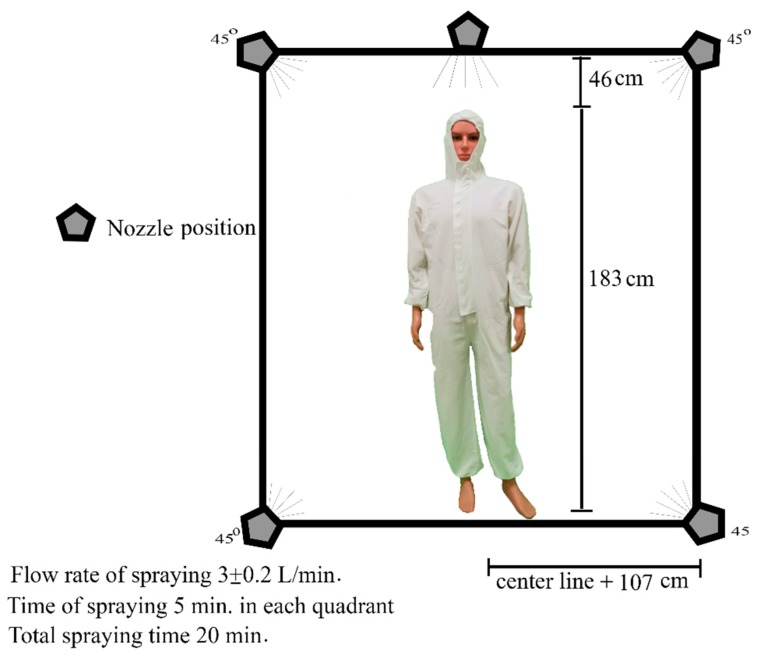
Closed chamber for experiment.

The efficiency of the gum rosin-coated PPC in its ability to protect against insecticides was tested in a closed chamber, modified from the ASTM F1359/F1359M−16a standard (Procedure A). The size of the closed chamber and position of the nozzles are shown in Figure 2.

Five nozzles were positioned in the same vertical plane, one at the top center above the clothed manikin, two nozzles at the upper sides, and two nozzles at the lower sides. The pressure for spraying the insecticides was set at 3 ± 0.2 L/min, spraying taking place simultaneously through all nozzles. The spraying time was set at 5 min in each quadrant from 0° to 360°, resulting in 20 min of total spraying time. The height of the manikin used was 183 cm, and it was positioned at the center of the chamber. Before testing, the opening of the hooded garment was sealed with waterproof tape.

### 2.4. Measurement of Protection Efficiency of the Gum Rosin-Coated PPC

Dermal exposure was measured using a pads technique. Potential dermal exposure (PDE) and actual dermal exposure (ADE) were measured. PDE is representative of the amount of insecticide that comes into contact with the body surface when the manikin is protected with PPC. ADE represents the total amount of insecticides that comes into contact with uncovered skin. 

Before the manikin was dressed in the coverall, 10 cm diameter pads of alpha cellulose backed with aluminum foil were attached to various locations of the manikin body for measuring PDE. The locations were as follows: head, neck, chest, back, upper arm, forearm, belly, genital area, upper leg, and lower leg [30]. External pads were also attached to the coverall at the same position as the internal pads for measuring ADE. At the end of the experiment, the pads were removed and analyzed to assess the concentration of the insecticide. The procedure was repeated 6 times for each piece of potential PPC. All pad samples were kept at −20 °C until chemical analysis.

### 2.5. Analysis of Insecticides

Chlorpyrifos (CAS Number: 2921-88-2) and Cypermethrin (CAS Number: 52315-07-8) were purchased from Dr. Ehrenstorfer GmbH (Augsburg, Germany). The alpha cellulose filter samples were extracted and analyzed using a modification of the method described by Sapbamrer and Hongsibsong [31] and Pakvilai et al. [32]. Twenty mL of acetonitrile (HPLC grade, J.T.Baker, Phillipsburg, NJ, USA) was used for paper extraction. This was shaken for 5 min in a 50-mL centrifuge tube. The alpha cellulose filter was repeated extract 2 times with 20 mL and 10 mL of acetonitrile. The extracted solutions were pooled and 3 g of sodium chloride (analytical grade, Fluka, Buchs, Germany) and magnesium sulfate (analytical grade, Fluka, Buchs, Germany) were added to remove the water. The combined solution was filtered through filter paper containing 2 g of anhydrous sodium sulfate (analytical grade, Fluka, Buchs, Germany) into 100 mL evaporating flask and evaporated to dryness using a rotary evaporator in a water bath at 40 °C. The flask was rinsed with 5ml ethyl acetate (HPLC grade, J.T.Baker, Phillipsburg, NJ, USA), transferred to a 15 mL glass tube, and dried with nitrogen. Finally, the residues were extracted using a 0.25 μm syringe filter and reconstituted in 1 mL of ethyl acetate for analysis by gas chromatography (GC).

The chlorpyrifos samples were analyzed using GC (Hewlett-Packard 7890 Series, Palo Alto, CA, USA). The apparatus was equipped with a flame photometric detector, a capillary column (DB-1701, 14% cyanopropyl-phenyl-methylpolysiloxane column—0.25 mm I.D. × 30 m length × 0.25 μm film thickness). Temperature was 250 °C for the injection port (spitless mode) and 250 °C for the detector port. The cypermethrin samples were also analyzed using GC (Hewlett-Packard 7890 Series), but the apparatus was equipped with an electron capture detector, a capillary column (HP-5, 5%-phenyl-methylpolysiloxane nonpolar column—0.25 mm I.D. × 30 m length × 0.25 μm film thickness). Temperature was 250 °C for the injection port (spitless mode) and 300 °C for the detector port. Temperature programming of the oven was as follows: initial temperature 100 °C for 1 min, first ramp at 5 °C/min to 180 °C (2 min), second ramp at 2 °C/min to 200 °C (1 min), third ramp at 5 °C/min to 280 °C (4 min), and final temperature maintained at 300 °C for 4 min. The carrier gas was helium 99.999% at 1.5 mL/min, constant flow mode. The total run time was 50 min.

### 2.6. Quality Control

The results of the quality control procedures are presented in Table 1. Limit of detection (LOD) and limit of quantification (LOQ) for chlorpyrifos were 0.020 μg and 0.025 μg, respectively. The LOD and LOQ for cypermethrin were 0.012 μg and 0.025 μg, respectively. Relative SD coefficient (% RSD) of chlorpyrifos was 5.7% for intra-batch and 3.0% for inter-batch, while the % RSD of cypermethrin was 6.1% for intra-batch and 7.6% for inter-batch. Recoveries ranged from 85.5 to 126.5% for chlorpyrifos, and 93.9 to 102.1% for cypermethrin.

### 2.7. Data Analysis

Surface area of each pad was 95.071 cm^2^, and ten pads were attached to various locations of the manikin body; therefore, total surface area of total pads was 950.71 cm^2^. Total surface area of the manikin body (except face, hands, and feet) was 19,130 cm^2^. Therefore, PDA and ADE for the total surface body of manikin were calculated by multiplying the amounts of insecticides in total pads by 20.12. The percentage of protection efficiency (% efficiency) to insecticides are as follows:

%efficiency = ((ADE−PDE) × 100)/ADE(1)

where

ADE = total amounts of insecticides from external pads

PDE = total amounts of insecticides from internal pads

Descriptive statistics, including mean and standard deviation (SD) were used. A one-way ANOVA (Tamhane’s T2 test) was used for testing the comparison of the protection efficiency to insecticides between the gum rosin-coated PPC and Tychem^®^ C coveralls.

## 3. Results 

### 3.1. Actual Dermal Exposure (ADE) and Potential Dermal Exposure (PDE) for the Total Surface Body of Manikin

ADE and PDE (ug) for the total surface body of manikin are presented in Table 2. ADE for chlorpyrifos and cypermethrin were 41,982.38 ± 6734.00 μg and 52,580.36 ± 7502.34 μg, respectively. PDE for chlorpyrifos ranged from 14.15 ± 7.67 μg for PPC1 to 6660.36 ± 6070.98 μg for PPC5. PDE for cypermethrin ranged from 56.01 ± 55.88 μg for PPC1 to 85,026.91 ± 29,890.51 μg for PPC5.

### 3.2. Protection Efficiency of the Gum Rosin-Coated PPC

Percentage of protection efficiency of each PPC are presented in Table 3. The results found that % protection efficiency for chlorpyrifos was 99.97 ± 0.02% for PPC1, 99.94 ± 0.04% for PPC2, 99.93 ± 0.12% for PPC3, 99.85 ± 0.23% for PPC4, and 84.14 ± 14.46% for PPC5. The proportion of protection efficiency for cypermethrin was 99.89 ± 0.11% for PPC1, 99.72 ± 0.21% for PPC2, 99.58 ± 0.55% for PPC3, 99.11 ± 1.42% for PPC4, and −61.71 ± 56.85% for PPC5. When we compared the protection efficiency of each PPC with Tychem^®^ C coveralls, the % protection efficiency for all PPC, except PPC5, was not significantly different to those for Tychem^®^ C coveralls. Interestingly, the highest level of protection efficiency was found in PPC1, followed by PPC2. 

## 4. Discussion

Our results found that the % protection efficiency for all PPC, except PPC5, was not significantly different to those for Tychem^®^ C coveralls. Interestingly, the highest level of protection efficiency was found in PPC1, followed by PPC2. These two fabrics were of twill weave construction and had the heaviest weight compared with other fabrics. It is likely that fabric construction and fabric weight were major factors affecting pesticide penetration through the garments. Our results are in agreement with a study by Shaw and Schiffelbein [19], which found that fabric weight and repellent finish were the significant factors affecting pesticide penetration. Previous studies also stated that other characteristics of fabrics such as thickness, yarn twist factor, cover factor, critical surface tension, and solid volume fraction, were significant parameters [18,21]. The PPC5 in our study had the lowest efficiency in protecting against chlorpyrifos and failed to protect against cypermethrin. This might be due to the low fabric count of the fabric and also its greater porosity when compared with other PPC (as shown in Figure 1). Therefore, our results suggest that choosing an appropriate fabric for coating with gum rosin is an important factor in the ability of a garment to protect against insecticides.

When comparing the protection efficiency among gum rosin-coated clothing and uncoated cotton clothing recorded in other studies, the efficiency of all PPC in our study, except PPC5, was higher than those of cotton fabrics from other studies. In a field study by Li et al. [33], total dermal exposure through cotton coveralls during the spraying of chlorpyrifos was investigated, and it was found that a single layer of cotton coveralls had effectiveness higher than 93%. A study by Gao et al. [34] also investigated operator exposure during spraying chlorpyrifos in maize fields, and they found that the protective efficiency of single layer clothes made of >70% cotton ranged from 16.9% to 68.1%, depending on the experience of the sprayers and the height of maize. Regarding previous studies in other chemicals, Protano et al. [9] estimated the performance of cotton garments for protecting against pesticides (including azinphos-methyl, terbutylazine, alachlor, dimethoate, and dicamba) during open field treatment. They reported that the protection efficiency conferred by cotton garments ranged from 84.1% to 92.5%. A laboratory study also found the protection efficiency of cotton fabrics against chemical agents ranged from 57.5% to 89.5% [23].

When comparing the protection efficiency with other water-repellent PPC and commercial PPC, the efficiency of all PPC in our study, except PPC5, had higher levels than those of the other PPC. A study by Protano et al. [9] mentioned that protection efficiency of pesticides given by the Tyvek coveralls range was approximately 97%. Similarly, Vitali et al. [35] investigated the same pesticides and claimed that the protection efficiency of specific protective garments, which were made of a synthetic material, was higher than 97.6%. A study by Thouvenin et al. [8] also investigated the protection efficiency against spinosad insecticides of different clothing during mixing/loading, application, and cleaning the equipment. The results showed that the protection efficiency was 95% for water-repellent finish polyester/cotton (65/35) coveralls and 98.7% for a CategoryIII Type3 partial body gown (Tychem^®^ F Gown style). However, these three studies were conducted in field situations which could result in under-estimation of the protective effects. In field situations, other factors might affect the outcome such as experience of sprayers, body movement of the workers, types of sprayer equipment, direction of spraying, plant characteristics, and environmental conditions [32,33,36,37]. 

With regard to previous studies in laboratory and field conditions, Espanhol-Soares et al. [24] investigated the efficiency of fluorocarbon finished fabrics in protecting against chemicals in both laboratory and field studies (copper hydroxide in laboratory conditions and sulfate manganese in field conditions). Beige fabrics (made with 100% cotton) and camouflaged fabrics (made with 69% cotton and 31% polyester) were coated with fluorocarbons to provide water repellence. The protection efficiency of the beige fabrics coated with fluorocarbon was approximately 96.8% for laboratory conditions and 95.8% for field studies. The efficiency of the camouflaged fabrics coated with fluorocarbon was approximately 93.9% in the laboratory and 97.1% in the field. The protection efficiency of this study was lower than that found in our study. This may be due to differences in the types of fabrics, test chemicals and experimental conditions. In addition, a study by Rahman Bhuiyan [23] also claimed that a polyurethane-aerogel incorporated coating on cotton fabrics had a protection efficiency of 100% for various chemicals. In vivo tests also evaluated the protective performance of different materials against carbamate insecticides. These included PU-coated nylon, water-oil repellent non-woven fabric based Sontara^®^, and Gore-Tex^®^ with Poly Tetra Fluoro Ethylene (PTFE). No pesticides residues were detected through the three fabrics, meaning that the protection efficiency of these fabrics was 100% [10]. The protection efficiency in our study is comparable to those reported by other studies in laboratory conditions. Therefore, it is reasonable to suggest that gum rosin-coated clothing provides satisfactory levels of insecticide protection, and it can be considered as suitable clothing for the protection against insecticides for pesticides applicators. 

A major outcome of this study is that the gum rosin-coated clothing was found to be an effective form of alternative clothing for agricultural workers to protect against insecticides because of its water-repellent characteristics, simple coating process, low cost, and raw material availability. Therefore, the gum rosin-coated clothing is suitable for agricultural workers who have limited or no purchasing power to access commercial PPC. They can use their own clothing of an appropriate material for coating with gum rosin. Furthermore, the coating process is simple, and agricultural workers could carry it out themselves in the future. However, allergic contact dermatitis from rosin contained in epilating product has been reported [38]. Therefore, the effects of the gum rosin-coated clothing and coating handlings on allergic contact dermatitis should be investigatedin further study. To add weight to the findings of this study, measurement of air permeability and other characterization of fabrics need to be carried out. Since this study investigated only first time use of the coated PPC, further tests involving multiple washes should be carried out [39]. Finally, a field study should also be carried out to explore the impact of other co-factors, such as body movement of agricultural workers and environmental conditions.

## 5. Conclusions

Gum rosin-coated clothing can be considered as suitable PPE to protect against insecticides in the case of pesticide applicators due to its water-repellent characteristics, simple coating process, low cost, and raw material availability. Choice of an appropriate fabric for coating with gum rosin was an important factor in the level of protection against insecticides. Due to its simple nature, sharing the availability of the knowledge of this process with pesticide applicators is feasible and achievable, leading to self-sufficiency in agricultural workers.

## 6. Patents

Coating process of fabrics with gum rosin followed the method described by Naksata M. and Naksata V. (Petty Patent no. 7450, 8 July 2010, Thailand, NSTDA, 2016). Naksata M. is a first author in this study.

## Figures and Tables

**Table 1:** Quality control for insecticide analysis.

Pesticide	LOD (µg)	LOQ (µg)	Correlation (*r*^2^)	Recovery %			Precision, % RSD	
				Low	Medium	High	Intra-Bath (*n* = 10)	Inter-Bath (*n* = 5)
Chlorpyrifos	0.020	0.025	0.99890	98.1	85.5	126.5	5.7	3.0
Cypermethrin	0.012	0.025	0.99945	93.9	102.1	98.2	6.1	7.6

**Table 2:** Actual dermal exposure (ADE) and potential dermal exposure (PDE) for the total surface body of manikin.

ADE/PDE		Chlorpyrifos(μg)		Cypermethrin(μg)	
		Mean ± SD.	Median	Mean ± SD.	Median
ADE	(*n* = 6)	41,982.38 ± 6734.00	41,828.42	52,580.36 ± 7502.34	54,711.38
PDE	PPC1 (*n* = 6)	14.15 ± 7.67	11.71	56.01 ± 55.88	37.47
	PPC2 (*n* = 6)	25.41 ± 16.58	24.99	148.13 ± 111.58	134.29
	PPC3 (*n* = 6)	29.76 ± 49.35	8.91	222.68 ± 290.48	160.88
	PPC4 (*n* = 6)	61.80 ± 98.31	27.83	468.16 ± 744.30	219.45
	PPC5 (*n* = 6)	6660.36 ± 6070.98	6642.88	85,026.91 ± 29,890.51	75,157.34
	Tychem^®^ C coveralls (*n* = 6)	21.36 ± 8.92	21.35	nd	nd

nd = not detected.

**Table 3:** Percentage of protection efficiency for chlorpyrifos and cypermethrin.

Types of Clothing	Chlorpyrifos			Cypermethrin		
	Mean ± SD.	Median	*p*-Value	Mean ± SD.	Median	*p*-Value
PPC1 (*n* = 6 ) ^a^	99.97 ± 0.02	99.97	<0.001 ^ae,be,ce,de,ef^	99.89 ± 0.11	99.93	<0.001 ^ae,be,ce,de,ef^
PPC2 (*n* = 6) ^b^	99.94 ± 0.04	99.94		99.72 ± 0.21	99.74	
PPC3 (*n* = 6) ^c^	99.93 ± 0.12	99.98		99.58 ± 0.55	99.69	
PPC4 (*n* = 6) ^d^	99.85 ± 0.23	99.93		99.11 ± 1.42	99.58	
PPC5 (*n* = 6) ^e^	84.14 ± 14.46	84.18		−61.71 ± 56.85	−42.94	
Tychem^®^ C coveralls (*n* = 6) ^f^	99.95 ± 0.02	99.95		100 ± 0	100	

^a^ = PPC1; ^b^ = PPC2; ^c^ = PPC3; ^d^ = PPC4; ^e^ = PPC5; ^f^ = Tychem^®^ C coveralls.
